# 

**Published:** 1892-01-23

**Authors:** 


					1IIK
FDR INFANTS
ISINGLASS
No. 278. Vol. XI.] j Saturday,
January 23rd, 1892.
[One Penny.

				

